# Developing a novel conceptual model of how food and drink tax policy impacts on consumption, health, environmental and economic outcomes

**DOI:** 10.1186/s12889-026-28589-2

**Published:** 2026-07-17

**Authors:** Penny Breeze, Amelia A Lake, Helen J Moore, Natalie Connor, Andrea Burrows, Katharine Pidd, Christian Reynolds, Rebecca Wells, Christina Vogel, Alan Brennan

**Affiliations:** 1 University of Sheffield, Sheffield, UK; 2 Teesside University, UK and Fuse – The Centre for Translational Research in Public Health, Middlesbrough, UK; 3 City St George’s, University of London,, London, UK; 4 MRC Lifecourse Epidemiology Centre, University of Southampton, Southampton, UK

**Keywords:** Fiscal policy, Public health, Simulation, Complex systems, Economic, Nutrition

## Abstract

**Background:**

While food taxes are proposed to promote healthier dietary choices and mitigate diet-related diseases, addressing rising UK obesity requires navigating a highly complex web of causal mechanisms. Economic evaluations of these interventions often lack a documented understanding of this systemic complexity. To bridge this gap, this study aims to comprehensively map the system by identifying the specific components affected, the causal pathways (mechanisms), and the individual and system-level factors (context) influencing the ultimate impact of food taxes.

**Methods:**

A diagrammatic representation of the conceptual model was developed through an iterative process. We first undertook a synthesis of reviews of food taxes and a rapid review of economic evaluations of food and drink taxes. The research team synthesised these results to describe mechanisms and outcomes for inclusion in the conceptual model. Secondly, the conceptual model was validated and revised according to feedback from 14 stakeholders across academia, policy, and third sector organisations.

**Results:**

Our final conceptual model illustrates system components which were grouped into eight sub-systems including policy infrastructure, industry behaviour, consumer behaviour, household expenditure, nutrition outcomes, health outcomes, environmental outcomes, and macroeconomic outcomes. Food taxes will influence consumption through price changes impacting purchases of taxed food and other purchases resulting in changes to consumption. Industry may modify the effects by absorbing the tax burden, marketing and product development and reformulation. We identify health, macroeconomic and environmental outcomes linked to food, and explore complex feedback loops linking health and macroeconomic performance to household finances further modifying food purchasing. We identify individual and contextual factors that modify these mechanisms.

**Conclusions:**

When studying the impacts of food and drinks taxes, researchers should consider the mechanisms by which individuals and industry can modify the effects of food taxes, and the extent to which these actions can be anticipated. System-wide factors can be documented so that the modelled evidence can be interpreted considering these factors even if they are not explicitly modelled. The conceptual model v3.0 remains dynamic and can be updated as evidence and perspectives on the food tax policy system develop over time.

**Supplementary Information:**

The online version contains supplementary material available at https://doi.org/10.1186/s12889-026-28589-2.

## Background

Dietary shifts towards food high in sugar, fats and/or salt and ultra-processed are contributing to non-communicable diseases and mortality [1]. Additionally, certain unhealthy foods cause significant ecological damage, so transitioning toward more nutritious eating patterns could be better for the environment and help meet national climate targets [2]. Socioeconomic factors also affect individual’s consumption of cheaper foods [3].

For the purposes of this study, we refer to food and drink as any substance consumed to provide nutrition, hydration and energy to humans. Alcoholic beverages are excluded from this work because the motivations for people to drink alcohol are distinct from food choices [4, 5]. In the United Kingdom (UK) most foods in retail settings do not incur value added taxes (VAT), whilst standard VAT is applied to some food groups and out of home venues. Since 2018 the Sugar Drinks Industry Levy (SDIL) has been applied to the production and import of soft drinks with more than 5 g sugar per 100 ml. Food taxes have been shown to improve dietary patterns by influencing consumer behaviour and encouraging healthier choices because price is a major driver of food choice [6, 7]. Previous analysis has shown that a salt and sugar tax on foods with added sugar and salt would lead to price rises, but would make those foods more expensive relative to healthier options, which could reduce purchases of unhealthy foods [8]. Taxes provide industry incentives for product reformulation of food products to improve the nutritional content of food as seen with the SDIL [9], and increases to government revenue, which can be allocated to other food policies such as free school meals.

Public health interventions operate within a complex system, and it is therefore necessary to understand how public health policies impact all aspects of the system [10]. It is not sufficient to consider only the expected effects on the parts of the system that are targeted by the policy. Policy evaluation should be embedded in a systems approach and aim to understand interactions with the environment and economy, which are important components of the food system [11]. Understanding how the system might impact the policy’s effectiveness and potential wider outcomes of the policy are also important components of complex systems evaluations [12, 13]. Food taxes are predominantly evaluated through empirical real-world evaluation [14, 15] or mathematical modelling [16, 17], both of which have been identified as suitable complex systems methodologies [18]. These methodologies are best executed when grounded in systems mapping processes. It is widely acknowledged that mapping how a policy interacts with a system to generate outcomes serves as an essential initial step, providing the necessary structural framework for subsequent complex systems evaluations [18–20].

This paper details a sub-study of a project situated within a broader initiative to identify food tax policies and quantitatively evaluate the outcomes of these policies by developing and adapting an existing model (HEALTHEI - NIHR133927) [21, 22]. We aim to develop our quantitative modelling by following the Squires et al. framework for developing the structure of public health economic models using a systems approach [19]. A systems approach to policy evaluation is necessary because food taxes do not operate in isolation. They trigger ripples across the supply chain and consumer behaviour that can either amplify or undermine health goals. By mapping these interactions, policymakers and modelers can better anticipate unintended feedback loops and provides a visual shared understanding of the system. A conceptual model provides a structured representation of the system for researchers to ensure that health economic evaluations are grounded in the complex reality of the food system, ultimately leading to more resilient and effective public health interventions. The specific objectives of this study were to undertake two evidence reviews to inform a conceptual diagram, run a series of workshops with academic experts and policy stakeholders, and finally to produce a consensus based conceptual model. This model documents the components of the food tax system, the mechanisms within the system that link policy change to outcomes, and the contextual factors affecting those relationships.

## Methods

**Fig. 1:**
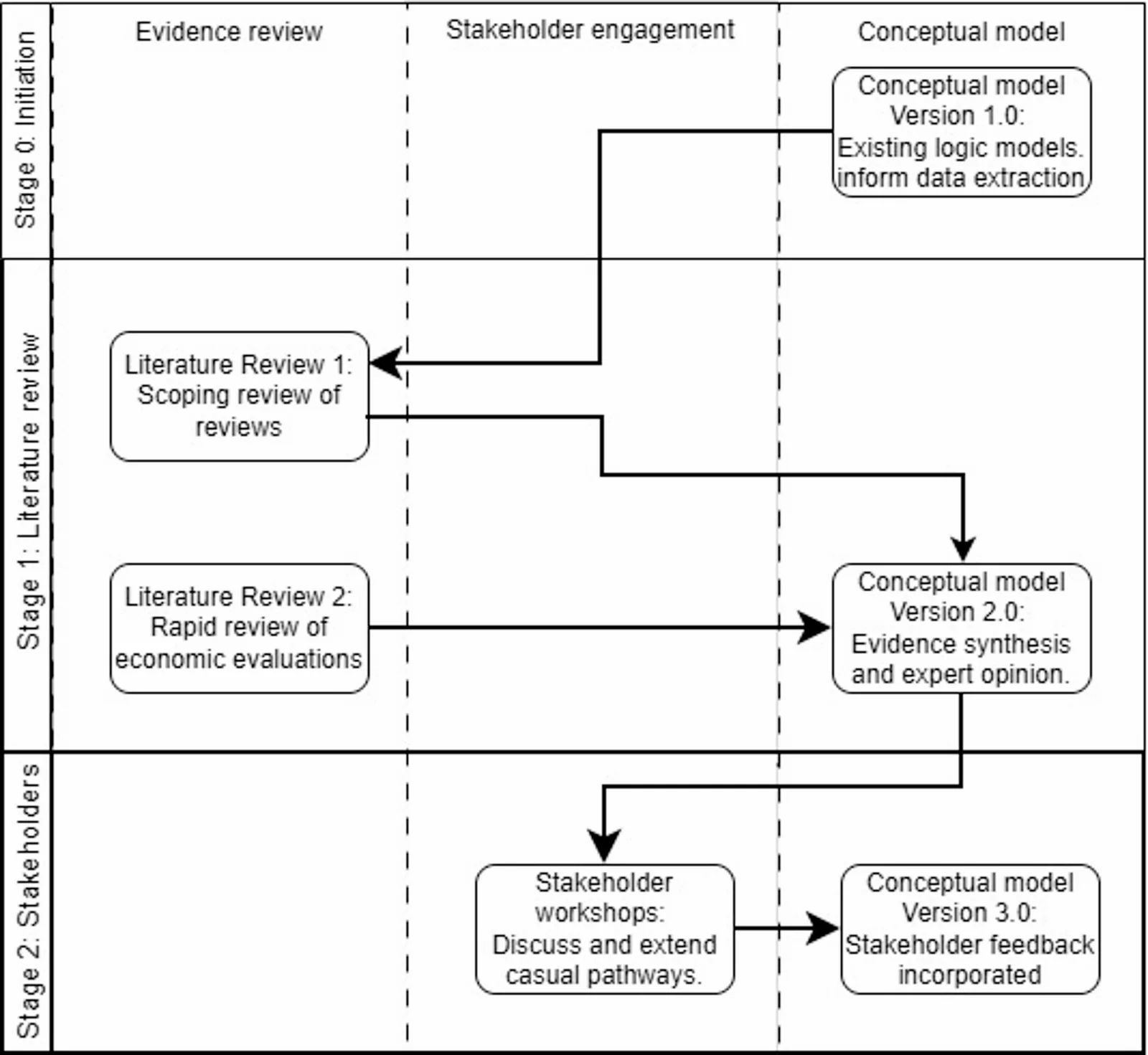
A flow diagram of methods used to iteratively develop the conceptual model

An iterative process was taken to create the conceptual model following a published framework for developing economic models for public health [19]. This framework was developed for public health economic evaluations, emphasising a systems-based approach to address the complexities of public health interventions. It guides modellers through a process to document an understanding the problem, engage stakeholders, and structure economic models. Figure 1 illustrates the stages of development and processes employed at each stage of this study. In the initiation stage, version 1.0 of the conceptual model was prepared to illustrate a preliminary understanding of the food tax system. We identified two diagrammatic representations/logic models of the food system to structure version 1.0 [23, 24]. The scope of these models was closely aligned with our proposed work; but neither was developed specifically for broad system description of food and drink taxes. One focused on health economic modelling for general food policy [23], while the other was specific to soft drinks [24]. Consequently, we used version 1.0 as a starting point to visualise the map, which was structured according to the themes that would direct the model’s subsequent evolution. The themes were derived from the HEALTHEI project’s aims to estimate the impact of food taxes on health, economic, labour market, environmental, household and inequalities. Version 2.0 of the conceptual model was informed by adding components, mechanisms, and contextual factors identified in two evidence reviews as well as expert opinions of the research team and their understanding of the food tax system (researchers PB, KP, AL, HM, CR, CV, RW, AB). Version 1.0 and 2.0 of the conceptual model are provided in the supplementary material: Appendix A. Workshop discussion with stakeholders were used to extend and refine the conceptual model. Contributions from stakeholders were incorporated into version 3.0 of the conceptual model. The HEALTHEI study protocol was registered in August 2022 [25].

### Evidence reviews

The literature reviews were used to develop version 2.0 of the conceptual model. Data extraction was conducted through an initial full-text review of all included articles by a primary reviewer. Each identified component, mechanism, and contextual factor that were related to the 5 themes, and those not already represented in the system were logged and categorized: components were mapped to specific sub-systems, while contextual factors were classified as either individual or macro-level variables. Data extraction was organised across five themes relating to health, economic, labour market, environmental, household and inequalities. To ensure a comprehensive review, the team opted to include all themes, acknowledging that version 1.0 did not fully capture the necessary breadth of the data, and did not adequately capture the complexity of the system. These themes included: (i) The mechanisms through which taxation translates to a change in consumption; (ii) The mechanisms linking food consumption to health; (iii) The mechanisms linking food consumption and health to non-health outcomes (environment, labour market, macroeconomic); (iv)The mechanisms linking household finances to the food system; (v) Contextual factors and individual characteristics.

For the first literature review, we synthesized international systematic reviews and discussion papers to establish a broad, interdisciplinary evidence base (Supplementary material: Appendix B) [26]. This approach deliberately transcended specific methodological or disciplinary boundaries to integrate both theoretical frameworks and real-world evidence. By accessing diverse sources, we aimed to identify and describe the multifaceted characteristics in the food tax system. In contrast to the broad disciplinary scope of the first review, we conducted a second rapid review [27], with a filter for health economic modelling studies to explore the technical boundaries of the models. The objective was to achieve a comprehensive understanding of the mechanisms, simulation structures, and outcomes utilised in studies with similar goals to our project. This review analysed food tax simulation studies from both high- and middle-income countries. (see supplementary material Appendix C).

### Workshops with stakeholders

Stakeholder engagement was sought to validate causal pathways based on expert experience, provide the informal logic missing from literature, and guard against the biases and disciplinary boundaries of the authors. Stakeholders from the UK were invited to attend group workshops. The facilitator guided discussion with participants through the following questions: (i) What components of the system are not represented in version 2.0 of the conceptual model? (ii) What mechanisms describing how the policy impacts the outcomes are not represented by the conceptual model? (iii) What demographic and social characteristics will affect the components and mechanisms of the model?

We hosted three online workshops in January-March 2023, to offer participants a choice of workshops and increase attendance. Participants who attended one of these three workshop were invited to attend a final workshop in person in October 2023. Participants were purposely selected to cover a mix of backgrounds. Participants from local authorities, national government departments, non-governmental organisations, and academia with an interest in food policy and food systems were invited to attend. Invitations purposively targeted experts with a range of expertise including health, nutrition, economics, and environment. The study was conducted according to the guidelines laid down in the Declaration of Helsinki and all procedures involving human subjects were approved by the Teesside University Psychology Research Ethics Sub-Committee, and all participants provided consent to participate.

Each participant was provided with a discussion document prior to the meeting. During the first three workshops version 2.0 of the conceptual model was displayed using interactive software to capture participants feedback and comments. The project lead (PB) presented the conceptual model, introducing the aims of the conceptual model, and provided details of how the conceptual model would be used to inform the later quantitative modelling work. Firstly, participants were asked to provide feedback on the conceptual model, through open discussion with other participants. Notes were taken to document these discussions. Secondly, all participants were asked to add comments to the online document. The conceptual model was grouped into sub-systems to aid presentation of the model and to allow participants to provide written comment at the sub-system level. The conceptual model was updated (by PB and KP) with feedback between the three online workshops to avoid duplication of comments. Finally, version 3.0 of the model was displayed in a poster on the wall at the final workshop (October 2023). All participants were provided with post-it notes and encouraged to add written comments and feedback on a printed posted version the diagram.

Following the workshop we extracted data from both facilitator notes and participant written feedback and classified this into components, mechanisms, and contextual factors if they related to the five themes for data extraction. Following the workshops, the included review articles were screened a second time to identify literature-based evidence supporting the additional insights provided by stakeholders.

### Analysis

We documented components, mechanisms and contextual factors in the system using a framework from Soft Systems Methodology [28] recommended by the Squires et al. framework [19]. In this approach the system is described as a set of interconnected components, and mechanisms as links between the components [28, 29]. The documentation informs a diagrammatic representation of the components and mechanisms to illustrate the conceptual model. Data analysis was documented in a narrative analysis of the components, mechanisms and contextual factors in the system. The arrows linking components represent relationships between components such that a change in one component is expected to impact on a change in another. The direction of arrows illustrates the mechanisms through which the policy impacts the system.

The study collected data from the synthesis of systematic reviews, rapid review, notes taken in the workshops, and written comments provided by participants at the workshops. The conceptual model was developed with a cascading approach in which each stage of the research contributed to the evolving conceptual model, i.e. data from workshop 1 was used to update the diagram presented in workshop 2. The data were integrated into the diagrammatic representation of the system if they were not already included. From this a narrative synthesis aimed to describe each component (Appendix D), mechanism and contextual factor. The conceptual model was used to identify feedback loops: closed chains of cause and effect where a change in one variable ripples through the system to eventually influence the original driver. To ensure internal validity, the diagram was presented to the broader research team at each stage of development for iterative critique and refinement.

## Results

**Fig. 2:**
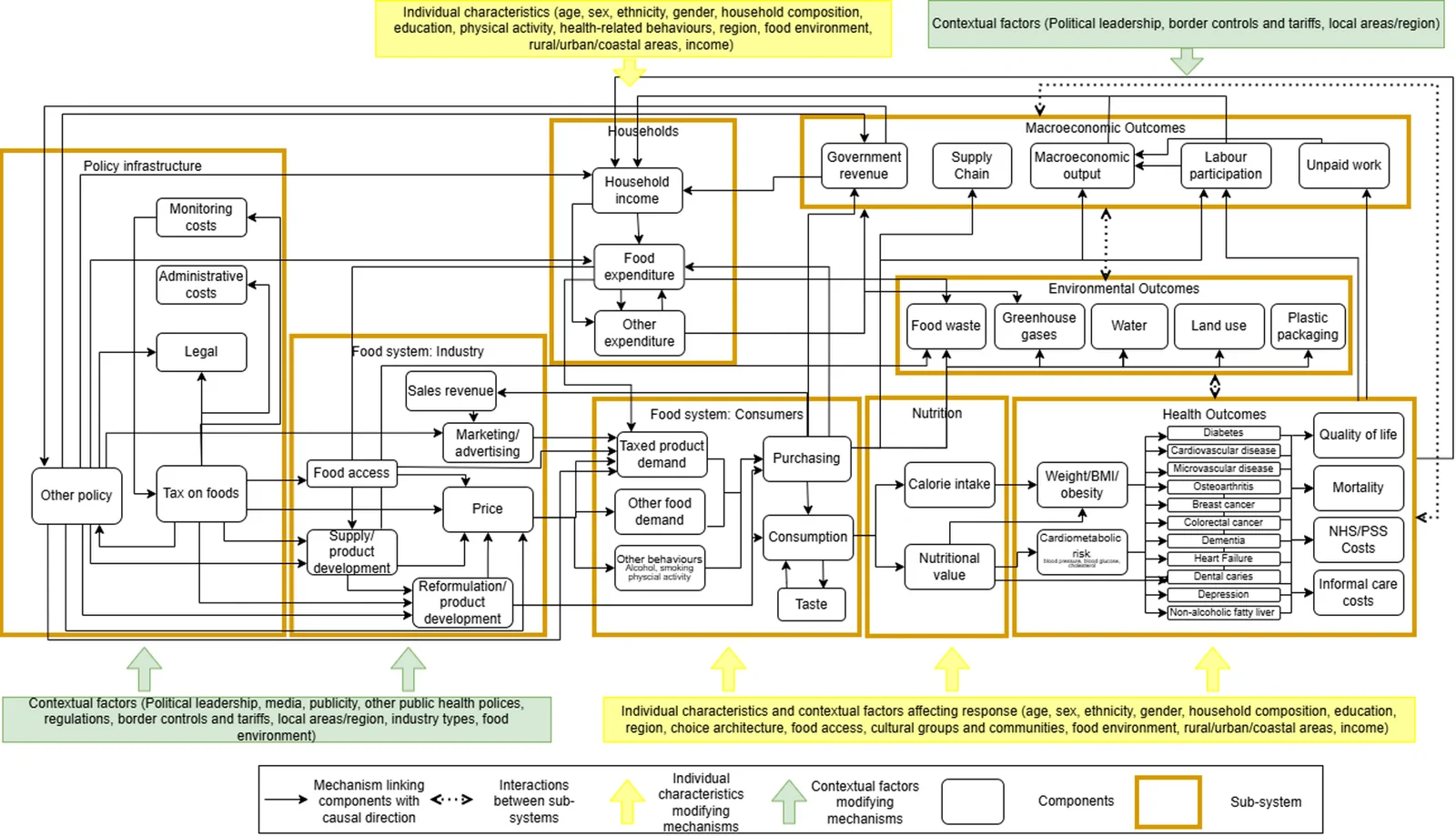
A conceptual model illustrating the mechanisms that food taxes will impact the wider system

**Fig. 3:**
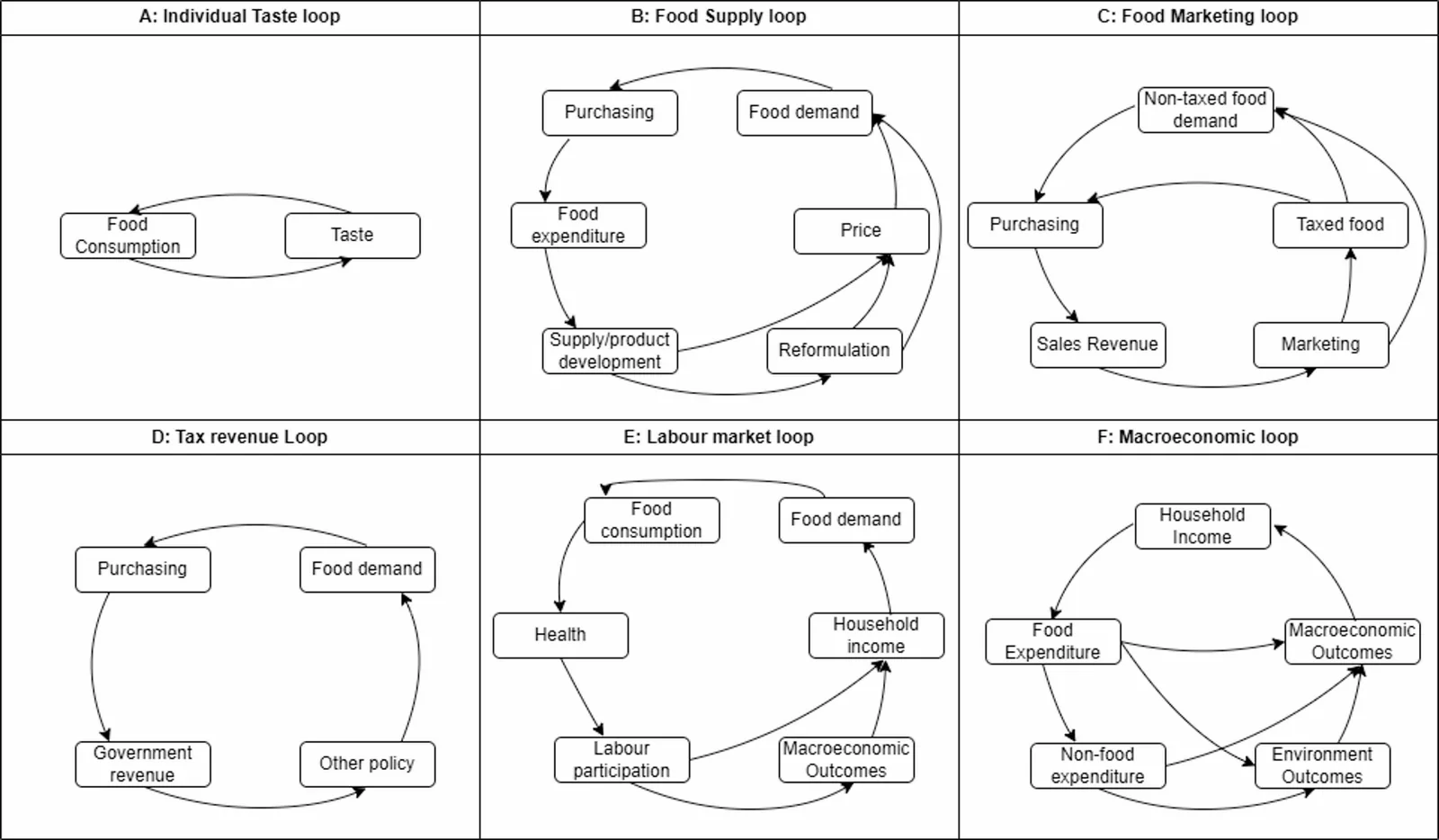
Feedback loops identified in the conceptual model

The synthesis of reviews identified 2,100 articles of which 32 eligible papers were identified. The rapid review of economic evaluations identified 2274 papers in the searches of which 21 met the inclusion criteria. A summary of each review and the findings are provided in supplementary material: Appendix B and C. Of 33 invitations distributed a total of 14 participants were available and consented to attend the online workshops. Participants were from non-government organisations (NGOs) (*n* = 9), academia (*n* = 3), the Civil Service (*n* = 1) and a local authority (*n* = 1). Of these 14 participants, nine also attended the in-person workshop in October. Key feedback from stakeholders expanded the contextual factors for the individual and system-level. The mechanisms through which consumers and industry respond to taxes were expanded to include feedback loops through marketing strategies, and consumer tastes. In addition, stakeholders highlighted the importance of reflecting the costs of food taxes within the policy infrastructure sub-system, and increased the number of environmental and macroeconomic outcomes. Figure 2 illustrates the final conceptual model (version 3.0). Figure 3 illustrates six feedback loops identified in the conceptual model.

### Conceptual model components

Within the conceptual model components were grouped into 8 sub-systems defined by PB to provide logical clusters that anchor specific components within an overarching complex system. *Policy infrastructure* refers to the national and local government actions to introduce and maintain food taxes and related policies. *Food system: Industry* refers to the behaviours and supply decisions of producers and retailers of foods. *Food system: Consumers*, refers to factors relating to the process of purchasing and consuming foods. *Nutrition* refers to the nourishment provided by the foods for health and growth. *Health Outcomes* refers to the status of an individual including diagnosed and non-symptomatic factors that contribute to an individual’s health. *Household outcomes*, refers to the consequences for a group of individuals who live together in a single dwelling. *Environmental Outcomes* describes the effects or results of actions, policies, or processes on the natural environment. *Macroeconomic Outcomes* refers to the overall functioning and performance of an economy at the national level. Interactions between sub-systems are highlighted to identify broad interdependencies between sub-systems. A description of each component is provided in the supplementary material: Appendix D.

### Conceptual model mechanisms

####  Food mechanisms

While food consumption is driven by taste, a reciprocal feedback loop exists: dietary shifts can gradually reshape taste preferences and lead to a positive or negative spiral that can reinforce changes in consumption and subsequently health, as illustrated in (Fig. 3A). The impact of a tax on changes in consumption is dependent on tax pass-through. Tax pass-through refers to the amount of a tax that is passed on to customers through a change in prices. Often it is assumed that 100% of a tax is passed onto customers, whereas lessons from historical data and other industries suggests that industry behaviour is more nuanced in their response [18, 24, 30, 31]. Tax pass-through may vary across food types [30]. Observations from the SDIL policy suggest that only 31% of taxes were passed on to customers, with other observations indicating changes in prices for non-eligible products and changes to product volume [9].

 Price elasticity of demand measures consumers response after a change in a product’s price [32–35]. Economic theory would suggest an overall rule that consumers buy less of a product when prices increase, with some exceptions. Economic theory provides a theoretical framework to understand the mechanisms in which price influences demand and estimates of the response to price are informative to understand the potential effects of taxes [35]. Consumers sensitivity to price varies across food products income, and ethnicity [32, 35, 36]. Empirical studies of the price elasticity of demand have been used extensively in the literature to estimate the impact of taxation on food purchases in modelling studies, and is supported by real world evaluations [24, 37, 38].

 Cross-price elasticity of demand measures the responsiveness of demand for other goods and services to a change in price of another product. Economic theory can be used to understand the responses [34]. Products can be described as either substitutes or compliments. Substitute products will increase demand due to an increase in price of another good, such as replacing an apple with a pear. In this instance, if the price of apples increases demand for pears increases. Complementary products will decrease demand in response to an increase in price for another product. Complementary products are often consumed together, such as breakfast cereal and milk, such that a price rise for cereals will decrease demand for milk. Cross-price elasticity of demand is important because preferences for substitute foods will impact on nutritional intake [33, 39–41].

Food taxes may lead to reformulation of food and product supply [18]. Firstly, manufacturers may use product “shrinkflation” to avoid changes in price. Secondly, manufacturers may modify existing recipes to reduce the tax burden [39, 41]. This pattern was observed in response to the UK SDIL policy, in which many drinks were reformulated to reduce sugar content [9]. Thirdly, manufacturers may be incentivised to increase the supply of healthier products to avoid the tax and decrease the supply of products incurring a tax [42]. As illustrated in Fig. 3B, there is a feedback loop between consumers and industry. While the industry may initiate reformulation, these changes may not be sustained if consumers respond negatively to the new products, as industry will be sensitive to consumer behaviour. These mechanisms introduce feedback loops through which the policy may have additional effects as the behaviour of consumers and industry iterates through incremental changes and responses which slowly emerge over time [43]. Therefore, effects through reformulation may be dynamic and unpredictable.

 Marketing and advertising are used by companies to promote food products to consumers, and influence consumer demand. Industries may use promotions and advertisements to offset loss of sales from price increases [18]. The visibility and public awareness of a tax may modify consumer responses to advertising campaigns [39]. The feedback loop in Fig. 3C suggests that food demand and industry marketing exist in a reciprocal relationship, where sales revenue provides the capital and incentive for industry to influence consumer purchasing through targeted promotion. Therefore, change in food demand in response to a tax policy may trigger marketing initiatives that modify future demand for food.

#### Health mechanisms

The conceptual model includes relationships for excess calorie intake and nutritional intake supported by a strong evidence base as a precursor to weight gain, body mass index (BMI) and obesity (Supplementary material: Appendix C).

 Cardiometabolic risks are impacted by nutritional intake. Therefore, it is important to acknowledge the effects of dietary change beyond weight and obesity. There may complementary or competing mechanisms within the wider nutritional effects on cardiometabolic risks [30, 44]. Changes to diet are complex and unhealthy foods are not necessarily substituted for healthy alternatives, for example chocolate substituted for crisps.

 Type 2 diabetes and cardiovascular diseases are strongly associated with BMI and cardiometabolic risks and were most commonly included as outcomes in the economic evaluations of food taxes [16, 17, 45–50] (see review of modelling studies in the supplementary material: Appendix C). The literature review identified that heart failure, osteoarthritis and cancers had also been linked to BMI in other economic evaluations. Dementia, non-alcoholic fatty liver disease, dental caries and depression were identified in only one review of food policy economic models [23].

Quality adjusted life years, healthcare costs and mortality are impacted by the health outcomes listed above. Workshop stakeholders highlighted the importance of social care costs and informal care costs of many of these conditions, and the increased cost of social care in individuals with multi-morbidities.

#### Mechanisms to environmental and macroeconomic outcomes

The conceptual model describes three main mechanisms in which macroeconomic outputs are impacted by food tax policy. Firstly, government revenues will be raised from food taxes [51]. This revenue can be used to fund health programmes or complementary food subsidies [37, 52, 53]. However, revenue may be volatile and estimates are usually imprecise [39]. Other policies funded by government revenue can shift consumer purchasing patterns, creating dynamic effects that ultimately influence future tax receipts, as illustrated in Fig. 3D. This can lead to a positive or negative spiral that can enhance or mitigate the improvements in food demand depending on the effectiveness of other policies. Secondly, food purchases contribute to Gross Domestic Product (GDP) [8]. The overall impact on GDP depends on the extent that household spending shifts to other goods and services [53]. Therefore, the industries contributing to food production and supply will be impacted by any changes in purchases through changes to sales revenue from increases/decreases in purchases [53]. Thirdly, the health status of the population is associated with labour participation and productivity, both in employment [53] and informal caring responsibilities.

There is a direct association between food production and environmental outcomes, such as greenhouse gases [46], water and land use [54]. Food is a driver of greenhouse gases through respiration of plants and animals, deforestation, and requires a large amount of water and land in agricultural processes. In addition, stakeholders highlighted that food waste and plastic packaging are linked to food purchases [55, 56]. These mechanisms vary between product types, with raw products using less plastic, but typically having shorter shelf-lives possibly leading to greater food waste [57].

#### Mechanisms between household and the food system

The conceptual model identifies that household finances are impacted by changes to health and the macroeconomic environment. Health outcomes impact household finances where illnesses require individuals to take sick leave, reduce working hours or shift to lower paid jobs. Similarly, macroeconomic output will impact household finances through employment conditions. Changes to household income may further impact food consumption, as budget constraints shift and lead to changes in consumer behaviours, as illustrated in (Fig. 3E). Substitution between food expenditure and non-food expenditure [48] may further impact the system by changes to environmental outcomes, macroeconomic outcomes, creating a system feedback loop illustrated in Fig. 3F [43]. The feedback loops can reinforce health benefits, for example if a healthier population have higher incomes to spend on fruit and veg. However, these loops may mitigate benefits if reduced macroeconomic output from the food sector reduces household finances leading to shifts to cheaper processed foods. The overall consequence of feedback loops are difficult to predict and will be sensitive to other dynamic factors, such as the waiting times for healthcare and inflation [39].

### Individual and system level contextual factors

We identified contextual factors to acknowledge that the mechanisms within the system are not homogenous, and that responses will vary according to these factors and characteristics [58].

The system-level factors mainly focussed on those that impact the implementation and sustainability of a food tax. We identified that taxes are more likely to be implemented with robust designs when accompanied by strong political leadership and positive support from public opinion and media [30, 39]. Publicity prior to the tax is important particularly if this focusses on the promotion of healthy alternatives [58–60]. Technical barriers to implementation can arise from legal challenges to government regulations, and conflicts with border controls and tariffs. Industry responses may depend on the industry types or size of business, and on their level of preparedness for the policy. Finally, food environment and food access differences will influence which populations can access to healthy alternatives and their flexibility to adapt to price changes. These impacts are likely to vary across local areas (urban/rural/coastal), and cultural groups within the UK.

Individual-level characteristics will influence the way that components are impacted by food taxes. Demographic factors such as age, sex (gender), household composition, and ethnicity are associated with patterns of food consumption, and health [36, 39, 61]. Socioeconomic factors such as education and income provide individuals with differential resources to adapt and respond to a food tax [62].

## Discussion

This novel study brings together a rich and emerging field of evidence to develop a conceptual model linking food tax policy to its outcomes with contributions and validation from an engaged group of expert stakeholders. This study attempted to take a broad overview of the food system, and identify the mechanisms through which food taxes impact individuals, households, healthcare, and the wider economic and environmental systems. This work also identifies the contextual features that interact with the mechanisms to modify the effects. The conceptual model does not aim to describe the quality of evidence for the mechanisms we have identified, but rather capture a comprehensive set of system components relevant to food taxes [19]. Key mechanisms within the conceptual model such as behavioural responses to price changes, and the effects of calories on weight gain, have a strong theoretical and evidence base. These mechanisms have been used extensively in modelling studies to evaluate food taxes. Other mechanisms in the conceptual model, such as linking health to unpaid care outcomes or household income, have weaker foundation in the reviewed evidence, and some mechanisms describe hypothesised causal relationships. We identified feedback loops within the system through which initial changes to the system (i.e. increase in price reduces consumption of unhealthy foods) may lead to secondary effects through further modification to industry behaviour (advertising campaigns to increase consumer demand), government revenue (financing new fruit and veg subsidies) or consumer behaviour (increased household income from health improvement increases spending on fruit and vegetables).

This study takes a complex systems approach to defining the conceptual model prior to economic model development. We have expanded on two existing logic models reporting the pathways through which food taxes would impact obesity [23, 24], and other health and non-health outcomes [23]. In other disciplines systems mapping exercises have aimed to understand the complex system of food systems focussing on supply chains, retailers and industry [63–65]. These systems maps highlight the range of pathways industry could adapt to policy change [63], and identify dynamic changes in food products responding to consumer demand for choice and drive to keep costs down [65]. This is consistent with our conceptual model in which we have identified multiple mechanisms through which industry could enhance or mitigate the health benefits of a tax.

This study advances existing literature by capturing the dynamic interplay of factors and identify key points of change in the system that can enhance or mitigate the policy effects. The conceptual model may provide insight into assessment of current barriers and enablers to change, actions that should be taken to overcome them, and possible consequences of doing so. Its practical application, however, is limited by current gaps in research regarding the causal directions between specific components. In addition, the conceptual model developed in this study can be used by researchers as a guide to the development of evaluations to design health economic models, inform the selection of outcomes and potential confounders for real-world evaluations. The conceptual model can be used to identify the strengths and limitations of fiscal policy evaluations across empirical and modelling study designs, which will assist policymakers in critically analysing the evidence prior to policy introduction. The conceptual model can be used as a tool to communicate the assumptions analysts make and highlight the wider context in which the analysis sits. This model might be particularly useful to explain findings that may be mitigated by unintended consequences and highlight areas for further data collection or research. Previous policy evaluation techniques often simplifying assumptions about the wider system either due to methodological or data limitations. The novel conceptual model could be used to communicate wider system effects to policymakers and other people affected by the food tax policy. Future iterations of the model should rank pathways by importance to focus research and policy interventions where they will have the greatest effect.

The conceptual model may be limited by stakeholder bias, and consultation with a range of different stakeholders may have resulted in a different focus or presentation of the conceptual model. Recruitment to the stakeholder workshops was challenging because many participants were not available to attend. Citizens and industry stakeholders were not invited to participate in the workshops. Their participation may have raised other factors for inclusion in the model, particularly those related to social or psychological factors. A more diverse consultative group could have shifted the focus or structure of the final model. Data extraction was systematically bounded by our five primary themes. While these themes were selected to capture a broad and diverse systemic landscape. We acknowledge that certain tertiary impacts, such as educational attainment, cognitive development, crime, and community cohesion, fall outside the current project’s scope. The model is focused on the UK context but the overall structure could be generalised to other jurisdictions because it is developed from international literature. However, it would benefit from a process of local validation and verification for use in other contexts. We would not characterise this conceptual model as a fixed output, but plan to use it as an evolving resource throughout the lifecycle of this project (NIHR133927) and beyond. The evidence base for fiscal food policies is rapidly emerging and the conceptual model needs to adapt to a dynamic social context to accommodate the ongoing effects of the cost-of living crisis and wider obesity policy.

In conclusion, we demonstrate the mechanisms by which individuals and industries might modify the effects of food tax policy change, and the extent to which these responses results in health and wider societal impacts. Evidence for food taxes can be interpreted in the light of the conceptual understanding of the system to interpret results from observational studies and add depth to the interpretation of modelling studies. We will use this conceptual model to inform the assumptions and data inputs to an evaluation of food taxes in the UK, set the modelling boundary, and interpret the finding of our analyses. 

## Supplementary Information

Supplementary Material 1.

## Data Availability

All data generated or analysed during this study are included in this published article and itssupplementary information files.

## Abbreviations

BMI: Body Mass Index
NGS: Non-governmental organisation
UK: United Kingdom
VAT: Value Added Tax
