# Supplementary material for: Developing a novel conceptual model of how food and drink tax policy impacts on consumption, health, environmental and economic outcomes
Source: BMC Public Health. 2026 Jul 17;26:3248. doi: 10.1186/s12889-026-28589-2 (PMC13741522; doi:10.1186/s12889-026-28589-2)
Supplement: Supplementary file 1 [file 12889_2026_28589_MOESM1_ESM.docx]

# Appendix A: Conceptual model versions

Figure S1: A conceptual model illustrating the mechanisms that food taxes will impact the wider system (version 1.0)

Figure S2: A conceptual model illustrating the mechanisms that food taxes will impact the wider system (version 2.0)

# Appendix B: scoping review of food and drinks tax reviews and discussion papers

Aims

To provide an overview of the impacts of food and non-alcoholic beverage price increases in national or local settings to facilitate the specification of an initial conceptual model for the evaluation of food taxes.

Method

The evaluation of tax and subsidy policies targeting food and non-alcoholic beverages is a growing area of research across public health, nutrition, and economics disciplines. We conducted a search in Pubmed/Medline between January 2010 and November 2021 to identify evidence for the impact and key issues on the system impact of price policies which targeted and non-alcoholic beverages on a broad range of health, economic and environmental outcomes. Search terms combined ***setting*** queries (fiscal, national, state, city, workplace, schools, supermarket, restaurant, fast food, and cafeteria), ***intervention*** queries (tax, subsidy, incentive, and price) and ***food*** queries (food, beverage, fruit, vegetable, soda, meat, dairy, fat, junk food, confectionery).

Literature reviews were included in the review of evidence if they identified and assessed evidence of food price policies on outcomes including food prices, consumption, health, economic or environmental impact. Full details of the inclusion and exclusion criteria are provided below.

|  | Inclusion | Exclusion |
| --- | --- | --- |
| Date | January 2010 | Before January 2010 |
| Language | English | Non-English |
| Location | All locations | No exclusions |
| Participants/Population | Human  Adults or children  All genders  All socio-economic backgrounds  All cultures | Selection by clinical diagnosis.  Restrictions by location i.e care homes |
| Intervention | Food duty or levy intervention imposed by government  Non-alcoholic beverage tax intervention imposed by government | Alcohol tax intervention  Food price rises imposes by non-governmental organisations.  Promotional product restrictions |
| Outcomes | Dietary intake measures (single food categories, dietary quality measures, dietary intake surveys, market research data)  Health  Economic  Environmental | No exclusions |
| Study design | Systematic reviews  Rapid reviews  Scoping reviews  Other non-systematic reviews | Original research i.e secondary data analysis of consumer data after tax |

Results

The search identified 2,100 articles published and a filter selected systematic reviews (N=67). Of the 67 studies identified 32 met the inclusion criteria. Figure S1 illustrates a PRIMSA diagram summarising the process of study selection.

Figure S1: PRISMA diagram for selection of studies into the review

**Included**

Included papers

(n=32)

Reports assessed for eligibility

(n=63)

Reports sought for retrieval

(n=63)

Records screened

(n=67

**Screening**

**Identification**

Records removed *before screening*:

Records marked as ineligible by filter for systematic review (n = 2033)

2,100 records identified from web of Science

Records excluded

Not related to food taxes (n=4)

Exclusion reasons:

- Ineligible intervention (n=8)
- Paywall / unavailable (n=1)
- Insufficient detail (n=1)

**Identification of studies via databases and registers**

The reviews summarised evidence from randomised controlled trials, real-world evaluations, price elasticity analyses, and simulation/modelling studies. A small number of systematic reviews (n=3) compared price-based policies with other dietary policy approaches and generally taxes/subsidies were found to be equally or more effective than other policies (1:3).

### The mechanisms through which taxation translates to a change in consumption of food

The price elasticity of demand for food varied from -0.44 for cheese to -1.08 for sugar sweetened beverages (5-7). The estimates highlighted that consumers are most likely to respond to price changes on meat, sugar-sweetened beverages, and take-aways. Price responses to sugar and confectionery were moderate, and lowest for high fat foods. There is a gap in the literature to describe price elasticity of demand for out-of-home food purchases.

A real-world evaluation of a Hungarian tax on sugar-added foods identified a 4% reduction in consumption of taxed foods (8). Reviews of real-world and intervention-based evaluations of taxes on SSBs concluded that these policies are effective in reducing SSB consumption (9:16). Real world evaluations of the Danish fat tax reported reductions in consumption across some high fat food groups (17), however challenges to the Danish fat tax resulted in it being repealed (18).

The mechanisms linking food consumption to health

Observed changes in health in response to a change in food and beverage prices are less common in real-world evaluations (18). There is limited evidence for the effects of SSB taxes on obesity (16). Experimental studies identify few studies reporting health outcomes (19:21). Reviews that have made the link between food price policies and health mostly rely on modelling studies (1, 13-15, 18, 24-26). These studies generally find that price policies lead to improvements in health (1, 13-14, 18, 24, 26). However, substitution to unhealthy foods reduce or reverse simulated health gains (23, 26).

The mechanisms linking food consumption and health to non-health outcomes

Only three reviews report the impact of food price policies on government revenue and wider macroeconomic consequences. Some studies estimate negative macroeconomic impacts on employment from implementing diet related fiscal policies (27). However, this evidence comes from industry sponsored reports and may be subject to bias. Diet-related fiscal policies are likely to raise revenue (26:27), but may be volatile over time as consumers adapt to price changes (18).

There is evidence that low income populations are more price responsive than high income populations and that price-based policies are likely to reduce health inequalities (28-30). The differences in price responsiveness between socioeconomic groups is likely to vary across food groups (5, 30) with more variation in responses to milk and take-aways (5).

The mechanisms linking household finances to the wider food system

Two studies discussed the dynamic ripple effects that taxes can have on industry, consumption and attitude that can have unpredictable effects on the long-term impact of fiscal policies (18, 32)

## References

1. Hyseni L, Elliot-Green A, Lloyd-Williams F, et al. Systematic review of dietary salt reduction policies: Evidence for an effectiveness hierarchy?. PloS one. 2017 May 18;12(5):e0177535.
2. Slapø H, Schjøll A, Strømgren B, Sandaker I, Lekhal S. Efficiency of In-Store Interventions to Impact Customers to Purchase Healthier Food and Beverage Products in Real-Life Grocery Stores: A Systematic Review and Meta-Analysis. Foods. 2021 May;10(5):922.
3. Hartmann-Boyce J, Bianchi F, Piernas C, et al. Grocery store interventions to change food purchasing behaviors: a systematic review of randomized controlled trials. The American journal of clinical nutrition. 2018 Jun 1;107(6):1004-16.
4. Gittelsohn J, Trude AC, Kim H. Pricing strategies to encourage availability, purchase, and consumption of healthy foods and beverages: a systematic review. Preventing chronic disease. 2017;14. E107.
5. Andreyeva T, Long MW, Brownell KD. The impact of food prices on consumption: a systematic review of research on the price elasticity of demand for food. American journal of public health. 2010 Feb;100(2):216-22.
6. Powell LM, Chriqui JF, Khan T, Wada R, Chaloupka FJ. Assessing the potential effectiveness of food and beverage taxes and subsidies for improving public health: a systematic review of prices, demand and body weight outcomes. Obesity reviews. 2013 Feb;14(2):110-28.
7. Cornelsen L, Green R, Turner R, et al. What happens to patterns of food consumption when food prices change? Evidence from a systematic review and meta‐analysis of food price elasticities globally. Health economics. 2015 Dec;24(12):1548-59.
8. Pfinder M, Heise TL, Boon MH, et al. Taxation of unprocessed sugar or sugar‐added foods for reducing their consumption and preventing obesity or other adverse health outcomes. Cochrane Database of Systematic Reviews. 2020(4).
9. Afshin A, Peñalvo JL, Del Gobbo L, et al. The prospective impact of food pricing on improving dietary consumption: a systematic review and meta-analysis. PloS one. 2017 Mar 1;12(3):e0172277.
10. Redondo M, Hernández-Aguado I, Lumbreras B. The impact of the tax on sweetened beverages: a systematic review. The American journal of clinical nutrition. 2018 Sep 1;108(3):548-63. https://doi.org/10.1093/ajcn/nqy135
11. Teng AM, Jones AC, Mizdrak A, Signal L, Genç M, Wilson N. Impact of sugar‐sweetened beverage taxes on purchases and dietary intake: Systematic review and meta‐analysis. Obesity Reviews. 2019 Sep;20(9):1187-204. https://doi.org/10.1111/obr.12868
12. von Philipsborn P, Stratil JM, et al. Environmental interventions to reduce the consumption of sugar‐sweetened beverages and their effects on health. Cochrane Database of Systematic Reviews. 2019(6). https://doi.org/10.1002/14651858.CD012292.pub2
13. Miracolo A, Sophiea M, Mills M, Kanavos P. Sin taxes and their effect on consumption, revenue generation and health improvement: a systematic literature review in Latin America. Health policy and planning. 2021 Jun;36(5):790-810. https://doi.org/10.1093/heapol/czaa168
14. Nakhimovsky SS, Feigl AB, Avila C, et al. Taxes on sugar-sweetened beverages to reduce overweight and obesity in middle-income countries: a systematic review. PloS one. 2016 Sep 26;11(9):e0163358. https://doi.org/10.1371/journal.pone.0163358
15. Backholer K, Sarink D, Beauchamp A, Keating C, Loh V, Ball K, Martin J, Peeters A. The impact of a tax on sugar-sweetened beverages according to socio-economic position: a systematic review of the evidence. Public health nutrition. 2016 Dec;19(17):3070-84. https://doi.org/10.1017/S136898001600104X
16. Itria A, Borges SS, Rinaldi AE, Nucci LB, Enes CC. Taxing sugar-sweetened beverages as a policy to reduce overweight and obesity in countries of different income classifications: a systematic review. Public Health Nutrition. 2021 Jul 5:1-27.
17. Lhachimi SK, Pega F, Heise TL, et al. Taxation of the fat content of foods for reducing their consumption and preventing obesity or other adverse health outcomes. Cochrane Database of Systematic Reviews. 2020(9).
18. Wright A, Smith KE, Hellowell M. Policy lessons from health taxes: a systematic review of empirical studies. BMC public health. 2017 Dec;17(1):1-4.
19. Alagiyawanna AM, Townsend N, Mytton O, Scarborough P, Roberts N, Rayner M. Studying the consumption and health outcomes of fiscal interventions (taxes and subsidies) on food and beverages in countries of different income classifications; a systematic review. BMC Public Health. 2015 Dec;15(1):1-4.
20. Adam A, Jensen JD. What is the effectiveness of obesity related interventions at retail grocery stores and supermarkets? a systematic review. BMC public health. 2016 Dec;16(1):1-8.
21. Naicker A, Shrestha A, Joshi C, Willett W, Spiegelman D. Workplace cafeteria and other multicomponent interventions to promote healthy eating among adults: A systematic review. Preventive medicine reports. 2021 Feb 23:101333.
22. Sawada K, Wada K, Shahrook S, Ota E, Takemi Y, Mori R. Social marketing including financial incentive programs at worksite cafeterias for preventing obesity: a systematic review. Systematic reviews. 2019 Dec;8(1):1-2.
23. Sisnowski J, Street JM, Merlin T. Improving food environments and tackling obesity: A realist systematic review of the policy success of regulatory interventions targeting population nutrition. PloS one. 2017 Aug 4;12(8):e0182581.
24. Niebylski ML, Redburn KA, Duhaney T, et al. Healthy food subsidies and unhealthy food taxation: A systematic review of the evidence. Nutrition. 2015 Jun 1;31(6):787-95.
25. Thow AM, Downs S, Jan S. A systematic review of the effectiveness of food taxes and subsidies to improve diets: understanding the recent evidence. Nutrition reviews. 2014 Sep 1;72(9):551-65.
26. Dodd R, Santos JA, Tan M, et al. Effectiveness and feasibility of taxing salt and foods high in sodium: A systematic review of the evidence. Advances in Nutrition. 2020 Nov;11(6):1616-30.
27. Mounsey S, Veerman L, Jan S, Thow AM. The macroeconomic impacts of diet-related fiscal policy for NCD prevention: a systematic review. Economics & Human Biology. 2020 May 1;37:100854.
28. Green R, Cornelsen L, Dangour AD, et al. The effect of rising food prices on food consumption: systematic review with meta-regression. BMJ. 2013 Jun 17;346.
29. Mizdrak A, Scarborough P, Waterlander WE, Rayner M. Differential responses to food price changes by personal characteristic: a systematic review of experimental studies. PloS one. 2015 Jul 7;10(7):e0130320.
30. McGill R, Anwar E, Orton L, et al. Are interventions to promote healthy eating equally effective for all? Systematic review of socioeconomic inequalities in impact. BMC public health. 2015 Dec;15(1):1-5.
31. Emmert-Fees KM, Karl FM, von Philipsborn P, Rehfuess EA, Laxy M. Simulation Modeling for the Economic Evaluation of Population-Based Dietary Policies: A Systematic Scoping Review. Advances in Nutrition. 2021 Apr 19.
32. Shemilt I, Marteau TM, Smith RD, Ogilvie D. Use and cumulation of evidence from modelling studies to inform policy on food taxes and subsidies: biting off more than we can chew?. BMC Public Health. 2015 Dec;15(1):1-8.

# Appendix C: Rapid review of modelling studies evaluating the health and economic impact of food taxes

## Aims

To identify modelling studies of food taxes and summarise the outcomes included in their methods.

## Method

The search was embedded within a broader rapid review of evidence for food taxes in high income countries. Screened all studies for modelling studies for inclusion in this review.

The search identified papers published between January 2010-December 2022 in a range of databases (PubMed, HMIC, Scopus, Google, Mintel/Mintel Food and Drink, Business Source Premier Ultimate). The search was restricted to high-income countries (defined by <https://data.worldbank.org/income-level/high-income>) and English language documents only.

Search terms: (food and beverages OR take-away OR junk food OR fast food OR sweet OR confectionery OR snack OR sodium OR salt) AND (tax OR price OR levy OR fiscal).

Inclusion criteria: Only studies of food taxes were included in the final data extraction. We restricted the inclusion to modelling studies in which outcomes are simulated by combining data from multiple sources of data to estimate a system change beyond consumption or nutrition in a mathematical model. Therefore, empirical analyses of single dataset to simulate changes in consumption or spending were not eligible for this review.

|  | Inclusion | Exclusion |
| --- | --- | --- |
| Date | Since 2010 | Before 2010 |
| Language | English | Non-English |
| Location | High income countries | Low and middle income countries |
| Participants | Human  Adults or children  All genders  All socio-economic backgrounds  All cultures | Selection by clinical diagnosis.  Restrictions by location i.e care homes |
| Intervention | Food duty or levy intervention imposed by government  Non-alcoholic beverage tax intervention imposed by government | Alcohol tax intervention  Food price rises imposes by non-governmental organisations.  Promotional product restrictions |
| Outcome | Any outcome measure  Objective or subjective dietary measures  Costs  Health outcomes (morbidity and mortality) | Opinion piece |
| Setting | Any setting | No restrictions |
| Study Design | Economic evaluations  Mathematical modelling | Mixed methods  RCT, Non randomised experimental  Case studies  cohort studies,  observational studies.  Primary Research  Systematic reviews |

Screening: All titles and abstracts were screened by two members of the WP1 team (NC,AB); their shortlist was reviewed by the research team. Full text versions of potential studies were assessed by members of the research team, consulting with another member of the WP1 team where any ambiguity existed. Conference proceedings/study protocols were categorised as an “ongoing study”. Reference lists of systematic reviews identified through the searches were also hand searched for potential studies. A second round of screening was performed to identify modelling studies meeting the inclusion criteria.

Extraction: A standardised data extraction template was developed. Evidence from the review was extracted by one reviewer under themes for model setting, model perspective, model structure, model outcomes.

## Results

The review identified 2274 papers, of which 67 were selected based in the first selection round without exclusion by study design. In the second round of study selection 21 studies of modelling studies meeting the inclusion criteria. Figure S2 illustrates a PRISMA flow diagram summarising the process of study selection.

**Figure S2: PRISMA 2020 flow diagram (55) for new systematic reviews which included searches of databases, registers and other sources**

**Identification of studies via other methods**

**Identification of studies via databases and registers**

2,274 records identified from:

- Medline (n=1,149)
- Scopus (n=934)
- Business Source Ultimate (n=149)
- HMIC (n=36)
- Mintel Food and Drink (n=6)

Records removed *before screening*:

Duplicate records (n = 547)

Records marked as ineligible by automation tools (n = 0)

Records removed for other reasons (n = 31)

Records identified from:

Websites (n = 100)

**Identification**

Records excluded

(n=1,342)

Records screened

(n=1,696)

Reports not retrieved

- Paywall (n=12)
- Language (n=5)
- Unavailable (n=5)
- Format (n=3)

Reports sought for retrieval

(n=363)

Reports not retrieved

(n=0)

Reports sought for retrieval

(n=100)

**Screening**

Exclusion reasons:

Duplicate (n=8)

Not relevant (n=71)

Inaccessible (n=4)

No available data (n=2)

Not modelling study (n=14)

Exclusion reasons:

- Ineligible outcome (n=85)
- Ineligible intervention (n=59)
- Not original research (n=58)
- Ineligible study design (n=37)
- Systematic Review (n=25)
- Paywall / unavailable (n=25)
- Foreign language (n=6)
- Ineligible country (n=4)
- Duplicate (n=1)
- Insufficient detail (n=1)
- Background article (n=1)
- Not Modelling study (n=32)

Reports assessed for eligibility

(n=338)

Reports assessed for eligibility

(n=100)

Included papers

(n=21)

**Included**

Economic evaluations commonly adopt cohort-based modelling structures, particularly for food tax evaluations, rather than individual microsimulation approaches. However, cohort models are much less flexible to look at the distributional impacts of policies across social groups, temporal changes of intervention effects, and estimating the timing of policy impacts. Economic evaluations commonly report the impact of policy on health and health-related cost outcomes, but much less frequently report the wider societal impacts on labour market and macroeconomic measures.

|  | Location/setting | Model structure | Perspective | Tax type |
| --- | --- | --- | --- | --- |
| Basu et al. (33) | United States | Individual level model | Healthcare | Sugar tax |
| Blakely et al. (34) | New Zealand | Multistate life table | Healthcare | F&V subsidy and junk food tax |
| Carter et al. (35) | Australia | Markov model | Employer | Unhealthy foods |
| Cobiac et al. (36) | Australia | Multistate lifetable | Healthcare | Saturated fat, sugar, and SSB |
| Ni Murchu et al. (38) | New Zealand | Multistate life table | Healthcare | Food taxes |
| Sacks et al. (39) | Australia | Multistate lifetable | Healthcare | Junk food tax |
| Smith-Spangler (40) |  |  |  |  |
| Zhang (41) | California | Agent-based model | No costs | Fast foods |
| Griffith (42) | England | Grouped cohort | Societal | Salt and sugar |
| Saha (43) | Sweden | Multistate lifetable | Healthcare | VAT reform |
| Smed (44) | Denmark | Multistate Lifetable | Healthcare | Fat tax |
| Harkanen (45) | Finland | Multistate Lifetable | Healthcare | Vat reform |
| Briggs (46) | United Kingdom | Multistate Lifetable | Health and Environment | Meat tax and SSB |
| Wilde (47) | United States | Comparative risk assessment | Healthcare | Meat and SSB |
| Briggs (48) | Ireland | Comparative risk assessment | Health | SSB |
| Gortmaker (49) | United States | Microsimulation | Healthcare | SSB |
| Levy (50) | United States | Comparative risk assessment | Not applicable | SSB |
| Pearson-Stuttard (51) | United States | Multistate lifetable | Healthcare | SSB |
| Lal (52) | Australia | Multistate Lifetable | Societal | SSB |
| Lin (53) | United States | Mathematical model | Not applicable | SSB |
| Goiana de Silva (54) | Portugal | Comparative risk assessment | Not applicable | SSB |

Table 1: Outcomes reported from modelling studies of food taxes

| Study | BMI/weight | Hypertension | Diabetes | CVD | Heart failure | Osteoarthritis | Cancer | QALY/DALY/HALY | Healthcare costs | Mortality | Productivity losses | Tax revenues | Economic output | Greenhouse gas emissions | Household expenditure |
| --- | --- | --- | --- | --- | --- | --- | --- | --- | --- | --- | --- | --- | --- | --- | --- |
| Basu et al. (33) | x |  | x |  |  |  |  |  |  |  |  |  |  |  |  |
| Blakely et al. (34) | x |  | x | x | x | x | x |  |  |  |  |  |  |  |  |
| Carter et al. (35) | x |  |  |  |  |  |  |  |  | X | x |  |  |  |  |
| Cobiac et al. (36) | x |  | x | x |  | x | x | x | x | x |  |  |  |  |  |
| Ni Murchu et al. (38) | x |  | x | x |  | x | x |  |  |  |  |  |  |  |  |
| Sacks et al. (39) | x |  | x | x |  | x | x | x | x | x |  |  |  |  |  |
| Smith-Spangler (40) |  |  |  | x |  |  |  | x | x | x |  |  |  |  |  |
| Zhang (41) |  |  |  |  |  |  |  |  |  |  |  |  |  |  |  |
| Griffiths (42) | X |  |  |  |  |  |  | X | X | X | X |  | X |  |  |
| Saha (43) | X | X |  | X |  |  | X |  |  | X |  |  |  |  |  |
| Smed (44) | X | X |  | X |  |  | X |  |  | X |  |  |  |  |  |
| Harkenan (45) | X |  | X | X |  |  |  |  |  | X |  |  |  |  |  |
| Briggs (46) | X | X |  | X |  |  | X |  |  | X |  | X |  | X |  |
| Wilde (47) | X |  | X | X |  |  |  |  |  | X |  |  |  |  |  |
| Briggs (48) | X |  |  |  |  |  |  |  |  |  |  |  |  |  |  |
| Gortmaker (49) | X |  |  |  |  |  |  |  | X | X |  |  |  |  |  |
| Levy (50) |  |  |  |  |  |  |  |  |  |  |  |  |  |  |  |
| Pearson-Stuttard (51) | x |  | x | x |  |  |  |  |  | x |  |  |  |  |  |
| Lal (52) | x |  | x | x |  |  | x |  |  | x |  | x |  |  | X |
| Lin (53) | x |  |  |  |  |  |  |  |  |  |  |  |  |  |  |
| Goiana de-Silva (54) | x |  |  |  |  |  |  |  |  |  |  |  |  |  |  |

## Summary

## References

33. Basu S, Lewis K. Reducing added sugars in the food supply through a cap-and-trade approach. American journal of public health. 2014 Dec;104(12):2432-8.
34. Blakely T, Cleghorn C, Mizdrak A, Waterlander W, Nghiem N, Swinburn B, Wilson N, Mhurchu CN. The effect of food taxes and subsidies on population health and health costs: a modelling study. The Lancet Public Health. 2020 Jul 1;5(7):e404-13.
35. Carter HE, Schofield DJ, Shrestha R, Veerman L. The productivity gains associated with a junk food tax and their impact on cost-effectiveness. PLoS One. 2019 Jul 22;14(7):e0220209.
36. Cobiac LJ, Tam K, Veerman L, Blakely T. Taxes and subsidies for improving diet and population health in Australia: a cost-effectiveness modelling study. PLoS medicine. 2017 Feb 14;14(2):e1002232.
37. Colchero MA, Paraje G, Popkin BM. The impacts on food purchases and tax revenues of a tax based on Chile’s nutrient profiling model. PLoS One. 2021 Dec 2;16(12):e0260693.
38. Ni Mhurchu C, Eyles H, Genc M, Scarborough P, Rayner M, Mizdrak A, Nnoaham K, Blakely T. Effects of health-related food taxes and subsidies on mortality from diet-related disease in New Zealand: an econometric-epidemiologic modelling study. PLoS One. 2015 Jul 8;10(7):e0128477.
39. Sacks G, Veerman JL, Moodie M, Swinburn B. ‘Traffic-light’nutrition labelling and ‘junk-food’tax: a modelled comparison of cost-effectiveness for obesity prevention. International journal of obesity. 2011 Jul;35(7):1001-9.
40. Smith-Spangler CM, Juusola JL, Enns EA, Owens DK, Garber AM. Population strategies to decrease sodium intake and the burden of cardiovascular disease: a cost-effectiveness analysis. Annals of internal medicine. 2010 Apr 20;152(8):481-7.
41. Zhang D, Giabbanelli PJ, Arah OA, Zimmerman FJ. Impact of different policies on unhealthy dietary behaviors in an urban adult population: an agent-based simulation model. American journal of public health. 2014 Jul;104(7):1217-22.
42. Griffith R, Jenneson V, James J, Taylor A. The impact of a tax on added sugar and salt. IFS Working Paper; 2021.
43. Saha S, Nordström J, Scarborough P, Thunström L, Gerdtham UG. In search of an appropriate mix of taxes and subsidies on nutrients and food: A modelling study of the effectiveness on health-related consumption and mortality. Social Science & Medicine. 2021 Oct 1;287:114388.
44. Härkänen T, Kotakorpi K, Pietinen P, Pirttilä J, Reinivuo H, Suoniemi I. The welfare effects of health-based food tax policy. Food Policy. 2014 Dec 1;49:196-206.
45. Smed S, Scarborough P, Rayner M, Jensen JD. The effects of the Danish saturated fat tax on food and nutrient intake and modelled health outcomes: an econometric and comparative risk assessment evaluation. European journal of clinical nutrition. 2016 Jun;70(6):681-6.
46. Briggs AD, Kehlbacher A, Tiffin R, Scarborough P. Simulating the impact on health of internalising the cost of carbon in food prices combined with a tax on sugar-sweetened beverages. BMC public health. 2015 Dec;16:1-4.
47. Wilde PE, Conrad Z, Rehm CD, Pomeranz JL, Penalvo JL, Cudhea F, Pearson-Stuttard J, O’Flaherty M, Micha R, Mozaffarian D. Reductions in national cardiometabolic mortality achievable by food price changes according to Supplemental Nutrition Assistance Program (SNAP) eligibility and participation. J Epidemiol Community Health. 2018 Sep 1;72(9):817-24.
48. Briggs AD, Mytton OT, Madden D, O’shea D, Rayner M, Scarborough P. The potential impact on obesity of a 10% tax on sugar-sweetened beverages in Ireland, an effect assessment modelling study. BMC public health. 2013 Dec;13:1-9.
49. Gortmaker SL, Wang YC, Long MW, Giles CM, Ward ZJ, Barrett JL, Kenney EL, Sonneville KR, Afzal AS, Resch SC, Cradock AL. Three interventions that reduce childhood obesity are projected to save more than they cost to implement. Health Affairs. 2015 Nov 1;34(11):1932-9.
50. Levy DT, Friend KB. Simulation modeling of policies directed at youth sugar-sweetened beverage consumption. American Journal of Community Psychology. 2013 Mar;51:299-313.
51. Pearson-Stuttard J, Bandosz P, Rehm CD, Penalvo J, Whitsel L, Gaziano T, Conrad Z, Wilde P, Micha R, Lloyd-Williams F, Capewell S. Reducing US cardiovascular disease burden and disparities through national and targeted dietary policies: a modelling study. PLoS medicine. 2017 Jun 6;14(6):e1002311.
52. Lal A, Mantilla-Herrera AM, Veerman L, Backholer K, Sacks G, Moodie M, Siahpush M, Carter R, Peeters A. Modelled health benefits of a sugar-sweetened beverage tax across different socioeconomic groups in Australia: A cost-effectiveness and equity analysis. PLoS medicine. 2017 Jun 27;14(6):e1002326.
53. Lin BH, Smith TA, Lee JY, Hall KD. Measuring weight outcomes for obesity intervention strategies: the case of a sugar-sweetened beverage tax. Economics & Human Biology. 2011 Dec 1;9(4):329-41.
54. Goiana-da-Silva F, Severo M, Cruz e Silva D, Gregório MJ, Allen LN, Muc M, Morais Nunes A, Torres D, Miraldo M, Ashrafian H, Rito A. Projected impact of the Portuguese sugar-sweetened beverage tax on obesity incidence across different age groups: A modelling study. PLoS medicine. 2020 Mar 12;17(3):e1003036.
55. Page MJ, McKenzie JE, Bossuyt PM, Boutron I, Hoffmann TC, Mulrow CD, Shamseer L, Tetzlaff JM, Akl EA, Brennan SE, Chou R. The PRISMA 2020 statement: an updated guideline for reporting systematic reviews. bmj. 2021 Mar 29;372.

# Appendix D: Description of all components in conceptual model v3.0

## Policy

### Tax on Foods

Taxes on foods refer to government imposed charges on the manufacture and or sale of food products. Taxes can be applied too all foods, or to a select sub-set of food types. Targeting foods that are assessed to be less healthy can generate a dis-incentive to purchase these products.

### Other Policy

Taxes on foods cannot be viewed in isolation from other government policies. Other policies will shape habits, cultural norms and attitudes to food, which in turn will impact on how food taxes impact on the food system. Furthermore, food taxes may impact the development and success of future food policies.

### Monitoring costs

These encompass the government expenses needed to track compliance and prevent tax evasion. Funding goes toward continuous food testing, grocery store audits, border inspections, and tracking supply chains to ensure products are correctly labelled and taxed.

### Administrative costs

These cover the internal operational expenses incurred by both authorities and businesses. Governments must fund tax collection infrastructure, while food businesses face overhead for updating point-of-sale systems and filing complex tax returns.

### Legal

Legal costs arise from resolving definitions, disputes, and enforcement actions. Money is spent on courtroom litigation over product classifications (e.g., defining a "biscuit" versus a "cake"), prosecuting fraudulent non-compliance, and drafting ongoing legislative amendments.

## Industry behaviour/supply

### Supply/product development

The food supply chain refers to the network of processes and activities involved in the production, processing, distribution and consumption of food. It includes agricultural production, processing, distribution, and retail. The supply of food impacts an individuals access to foods due to the availability of retail and out of home outlets.

### Marketing/advertising

Marketing is the overarching strategy and process of brings a product to market. Marketing encompasses a broad range of activities aimed at identifying, creating and satisfying customer needs. It aims to understand consumer behaviour to develop products, set prices, distribute products, and creating strategies to communicate the value of products and encourage sales. Advertising is a specific component of marketing focussed on promoting products to customers through various channels.

### Reformulation/product development

Reformulation refers to the process of altering the composition of a food product to change its nutritional profile. This might be in response to industry pressures, consumer preferences, or regulatory requirements. The process can involve changes to the ingredients used in order to alter the nutritional value of the product, or changes to the size of the product. Product development refers more specifically to the introduction to the market of new products, which may marketed a distinct products rather than reformulations of existing products.

## Consumer Behaviour/demand

### Taxed product demand

The amount of the taxed food that individuals wish to purchase.

### Other food demand

The amount of other foods that individuals wish to purchase.

### Other behaviours (alcohol, smoking, physical activity)

Demand for food is correlated with the demand for other health behaviours. Increases in physical activity may impact the demand for food by increasing energy expenditure. Smoking is known to be an appetite suppressant. Alcohol consumption may impact consumers tastes and preferences for food.

### Purchasing

Purchasing refers to the process of acquiring foods from external sources to meet individual or household needs. For the purpose of this conceptual model purchasing is limited to the acquisition of foods in exchange for money.

### Consumption

Consumption refers to the process by which individuals use foods to satisfy their needs. In contrast to purchasing consumption only includes foods that are eaten or drunk, so excludes any food that is wasted or disposed of. For an individual it may also include foods that are acquired by other means than purchasing, such as food purchased by a third party.

### Taste

Taste is the sensory perception of five basic tastes, driven by chemical interactions that trigger biological adaptations based on the foods we frequently consume.

## Nutrition

### Calorie intake

Calories are units of energy that the body uses for maintaining body function, physical activity and growth. Calorie intake refers to the total number of calories consumed through eating and drinking over a specified period. Managing calorie intake is crucial for maintaining, losing or gaining weight.

### Nutritional value

Nutritional value refers to the balance of nutrients that are included in individual food items and a persons diet. Nutritional value describes the amount, or proportion, of diet that includes carbohydrate, fat, protein, vitamins and minerals. The nutritional value is provided on food labels.

## Health Outcomes

### Weight/Body Mass Index (BMI)/obesity

Body size can be measured in a number of ways, but the most commonly used is weight, and body mass index (BMI), a numerical value that is derived from an individual’s height and weight. BMI can be assigned to categories based on the numerical value and can be used to assess whether a person has a healthy body composition. BMI is known to have limitations in indicating health, because it does not assess whether the weight is stored as fat. However, on average high BMI has been associated with poorer health.

### Cardiometabolic risks (blood pressure, blood glucose, cholesterol)

Individuals at risk of cardiovascular disease may have a cluster of risk factors including obesity, raised blood pressure, high blood glucose, abnormal lipids as well as abdominal obesity. Population studies have identified strong correlation between the indicators of this risks, suggesting the potential for a common aetiology of these risks.

### Diabetes

Type 2 diabetes is a condition that causes too much sugar in your blood. It can cause serious health problems if not treated. Type 2 is the most common form of diabetes, and in contrast to type 1 diabetes is less likely to be diagnosed in childhood, and can be prevented or managed with lifestyle changes.

### Cardiovascular disease

Cardiovascular disease refers to a broad range of disease that relate to the circulatory system. The complications of cardiovascular disease can include coronary heart disease, heart failure, and stroke.

### Microvascular disease

Microvascular diseases refer to a type of non-obstructive coronary artery disease that causes the small blood vessels feeding the heart muscle to not work as they should. Here we refer to those microvascular diseases commonly associated with type 2 diabetes including retinopathy, neuropathy, and nephropathy.

### Osteoarthritis

Osteoarthritis is a degenerative joint disease, in which the tissues in the joint break down over time. It is the most common type of arthritis.

### Breast cancer

Breast cancer is a disease in which abnormal breast cells grow and form tumours. Without treatment breast cancer tumours can spread throughout the body and can lead to mortality.

### Colorectal cancer

Colorectal cancer is a disease in which abnormal cells grow in the colon inner lining and form tumours. Without treatment breast cancer tumours can spread throughout the body and can lead to mortality.

### Dementia

Dementia is a collection of diseases that affect cognitive function and memory leading to a reduction in an individual’s ability to carry out daily functions.

### Heart Failure

Heart failure means that the heart is unable to pump blood around the body properly.

### Dental caries

Dental caries refers to a form of tooth decay in which there is damage to the enamel surface of the tooth. This damage can lead to cavities that require tooth extraction.

### Depression

Depression is a mental health condition affecting mood. Depression can range from mild symptoms to very severe, leading to a range of symptoms and impacts on daily activities.

### Non-alcoholic fatty liver disease (NAFLD)

NAFLD refers to conditions caused by the build up of fat in the liver. NAFLD is often split into stages from a mild form that does not have major health implications to liver failure.

### Quality of life

Quality of life (QoL) and health-related quality of life (HRQOL) are multi-dimensional concepts commonly used to examine the impact of health status on quality of life. It is often measured by asking questions across a number of dimensions that are associated with individuals quality of life thar can be impacted by health status. In health economics several measures of HRQoL are used, bwith the EQ-5D being preferred in the UK.

### Mortality

Mortality refers to a health state of death. It refers to the point in time of death, and can be used to estimate the number of years of life lived, and changes to mortality can be used to quantify and improvement in health outcomes.

### National Health Service and Personal Social Services (NHS/PSS) Costs

The costs incurred by the National Health Service to deliver health and social care services. The categories of cost included in analyses from the NHS and Personal Social Services perspective were hospital care, community-based health care, community-based social care and medication use.

### Informal care costs

Informal care costs refer to the costs of social care that are not publicly funded.

## Environmental outcomes

### Food waste

Food waste refers to food that is uneaten, and can be discarded at various stages of the food supply chain. This can be due to inefficiencies in farming, losses due to production techniques, excess stocking in retain environments, or over purchasing in households.

### Greenhouse gases

Atmospheric gases that trap heat from the sun, thereby warming the Earth’s surface through the greenhouse effect. Major greenhouse gases include carbon dioxide, methane, nitrous oxide, fluorinated gases. Food is a driver of greenhouse gases through respiration of plants and animals, and deforestation.

### Water

Food production requires a large amount of water use in the agricultural processes. Agriculture is the largest consumer of water globally, primarily for irrigation of crops and watering of livestock. Over extraction of water resources can lead to depletion of the water supply, and climate change. Agricultural process can also lead to contamination of water.

### Land use

Land use refers to the management and modification of natural environment or wilderness into built environments such as field, pastures, and settlements. Deforestation for agriculture results in a loss of biodiversity and carbon sequestration capacity.

### Plastic packaging

Plastic packaging is widely used across the food industry, and involves significant use of fossil fuels and energy. Plastic is a significant contributor to waste, with plastic packaging ending up in landfill or as litter. Plastic wates can also end up in oceans and rivers harming marine life.

## Macroeconomic outcomes

### Labour participation

Labour participation refers to the proportion of a country’s working-ae population that is either employed or actively seeking employment. Economic inactivity can be driven by a number of reasons including, caring responsibilities, ill health, job availability, or participation in the informal economy. It is an indicator of the health of the working society.

### Macroeconomic output

The total value of goods and services produced by an economy over a specified period. In is an indicator of economic health, and is used to assess the performance and growth of the economy. An example of macroeconomic indicators are gross domestic product (GDP).

### Supply chain

A supply chain involves the processes and entities involved in producing and delivering a product or service to the end customer. It includes the raw materials used in production to final product delivery.

### Government revenue

Government revenue refers to the funds that a government collects to finance government services and operations. In this context government revenue is mainly focused on taxes as an important source of government revenue. Taxes can be applied to the sales of goods and services either as a proportion of price, or targeting a specific product.

### Unpaid work

Domestic labour (cooking, cleaning) and caregiving (parenting, eldercare) sustain the current and future workforce, directly supporting individual well-being. Additionally, community volunteering strengthens social safety nets and drives non-profit sectors. Formal economic systems rely on unpaid contributions but these are rarely accounted for in Gross Domestic Product calculations.

## Household outcomes

### Household income

The combined income of all members of a household over a specified period. Income can be from employment, welfare benefits or financial gains.

### Food expenditure

The amount of money spent by a household over a specified period. The proportion of a household income will be spent on food, and the proportion is likely to be highly related to the total income of the household.

### Other expenditure

Expenditure on all non-food items and services. This can include expenditure on other commodities, such as tobacco and alcohol, but also include spending on housing, energy bills etc…

# Appendix E: Feedback loops highlighted in conceptual model v3.0

Figure S3: A conceptual model highlighting six feedback loops within final conceptual model (version 3.0)
